# Response to: norepinephrine formulation for equivalent vasopressive score

**DOI:** 10.1186/s13054-023-04404-x

**Published:** 2023-03-28

**Authors:** Yuki Kotani, Giovanni Landoni, Alessandro Belletti, Ashish K. Khanna

**Affiliations:** 1 Department of Anesthesia and Intensive Care, IRCCS San Raffaele Scientific Institute, Via Olgettina 60, 20132 Milan, Italy; 2 School of Medicine, Vita-Salute San Raffaele University, Via Olgettina 58, 20132 Milan, Italy; 3 Department of Intensive Care Medicine, Kameda Medical Center, 929 Higashi-Cho, Kamogawa, Chiba 296-8602 Japan; 4 Department of Anesthesiology, Section On Critical Care Medicine, Wake Forest Center for Biomedical Informatics, Perioperative Outcomes and Informatics Collaborative, Medical Center Boulevard, Wake Forest University School of Medicine, Winston-Salem, NC 27157 USA; 5 Outcomes Research Consortium, Cleveland, OH 44195 USA

Dear Editor,

We thank Mongardon et al. for their interest in our manuscript on an updated norepinephrine equivalent score in intensive care [1]. They correctly pointed out that two formulations of norepinephrine (2 mg/mL and 1 mg/mL) are available in different countries and hospitals [2].

Extensive research in drug agency databases and contacts with colleagues in five continents surprisingly showed that norepinephrine (better defined as norepinephrine base) is unavailable in the market. Each country has one or more commercially available products, which are different salt formulations, named tartrate, bitartrate, hemitartrate, hydrochloride, or maleate. On average, norepinephrine salts seem to be half potent as norepinephrine base (although pharmacists would consider it as a matter of dilution, not potency). Even if these issues have existed in the last decades, they have not been addressed properly.

Since expert physicians carefully and judiciously titrate norepinephrine with a blood pressure target, the dose is patient-tailored in most situations. It is also true that initiation of a second- and third-line vasoconstrictor, use of off-label vasoconstrictors, and administration of mechanical circulatory support might be triggered by the administered dose as expressed by µg/kg/min. It is unknown whether the experts writing the guidelines, commentaries, and existing vasoactive equivalent scores were referring to norepinephrine base, which does not exist and has never existed, or norepinephrine salts. From the pharmacological point of view, it would be correct to expect that the drug is prepared by pharmacists and nurses worldwide according to norepinephrine base dose equivalence, and therefore, this is what guidelines and existing vasoactive scores are likely referring to. At the same time, situations are likely very jeopardized globally between different intensive care units and hospitals in the same country using different doses, either norepinephrine base or less potent (more diluted) norepinephrine salts.

Multidisciplinary and international consensus concerning the formulation used to describe norepinephrine dose would help prevent dosing confusion in daily practice and facilitate interpreting clinical trial findings in different settings.

## Data Availability

Further information is available from the corresponding authors upon reasonable request.
